# An *in vitro* study on the effect of an oscillating stripping method on enamel roughness

**DOI:** 10.1186/s40510-014-0071-8

**Published:** 2015-02-10

**Authors:** Stefan Baumgartner, Anna Iliadi, Theodore Eliades, George Eliades

**Affiliations:** Clinic for Orthodontics and Paediatric Dentistry, Center of Dental Medicine, University of Zurich, Plattenstr. 11, 8032 Zürich, Switzerland; Department of Dental Biomaterials, School of Dentistry, University of Athens, 2 Thivon Str, 115 27, Goudi Athens, Greece

**Keywords:** Stripping, Enamel roughness, 3D profilometry

## Abstract

**Background:**

The aim of the present study was to assess the changes in enamel roughness parameters before and after stripping with an oscillating diamond strip system.

**Methods:**

Sound premolars extracted for orthodontic reasons were embedded up to their cervical area in a polyvinylsiloxane putty, creating four groups of four teeth with three interproximal areas each (mesial/distal). The same regions of interproximal enamel surfaces were studied by 3D optical interferometric profilometry before and after stripping with the Ortho-Strips system (Intensiv Dental SA, Montagnola, Switzerland) (*n* teeth = 16, *n* contact points = 12, *n* sites measured = 24). The roughness parameters tested were the amplitude parameters Sa and Sz, the hybrid parameter Sdr, and the functional parameters Sci and Svi. The parameter differences (ΔSa, ΔSz, ΔSdr, ΔSci, ΔSvi) were calculated per region and statistically analyzed by one-sample Mann-Whitney rank sum test (*a* = 0.05).

**Results:**

High statistically significant differences were found in ΔSa, ΔSz, and ΔSvi median values (0.453, 3.870, and 0.040 μm, respectively); a significant difference in ΔSdr median value (1.514%); and no statistically significant difference in ΔSci (0.110 median value).

**Conclusions:**

Under the conditions of the present study, the Ortho-Strips system seems to significantly increase the amplitude parameters Sa and Sz; the hybrid parameter Sdr, associated with the developed interfacial area ratio; and the functional parameter Svi, which depicts the volume of the deepest valleys. Under the conditions of the present study, the Ortho-Strips seems to significantly increase four of five measured roughness parameters.

## Background

Interproximal stripping, defined as the removal of interproximal enamel, is routinely used in orthodontics to eliminate Bolton tooth-size discrepancies between teeth of the maxillary and mandibular anterior and posterior arch [1,2], treat mild or moderate crowding [3], prevent relapse [4,5], and improve aesthetics [5]. This procedure, also known as interdental enamel reduction, interdental polishing, enamel approximation, or slenderizing [6], may have a favorable effect on treatment duration and allows transverse arch dimensions and anterior inclinations to be maintained.

Stripping may have a variable extent, and although in most cases it entails a 1- to 2-mm mesiodistal reduction, removal of as high as 50% in enamel width has been reported, which in radical cases can generate 7 mm of space [7]. If the anterior dentition also is included, an additional gain of 2.5 mm can be achieved [8]. At the moment, most authors suggest about 0.3 to 0.5 mm per tooth surface as the maximum amount of stripping without severely affecting tooth structure [9], or up to half of the enamel thickness [8]. It has been recommended that reduction should not exceed 0.3 mm of the width of maxillary incisors, 0.6 mm in maxillary premolars and molars, 0.2 mm in the mandibular incisors, and 0.6 mm in the mandibular premolars and molars [10].

Despite its advantages, enamel reduction has attracted the concern of investigators, owing to enamel tissue removal. In operative dentistry, it is crucial to maintain contact points in the posterior teeth to allow for proper self-cleaning and avoid the retention of plaque, although in orthodontic treatment, the interdental tooth enamel is ground down therapeutically. Adequate polishing of the stripped teeth is essential for a good long-term prognosis, since scratches and furrows remaining on the enamel surface facilitate plaque accumulation and possibly caries development [11-13]. A number of researches have focused not only on the orthodontic aspects but also on the cariogenic and periodontal implications associated with this procedure since it has been shown that changes in enamel integrity may be considered as a predisposing factor for caries and periodontal disease [11,12]. After grinding, the tooth surface seems to become rougher, with a significantly greater plaque accumulation capacity [3,12,14,]. Although the results of a recent systematic review [15] were inconclusive regarding enamel roughness after interdental stripping *in vitro*, the meta-analysis of the *in vivo* results did not find that interdental enamel reduction was a predisposing factor for caries development.

Proximal enamel reduction entails a wide array of mechanical, automatic, rotating, or translatory devices. Recently, oscillating mechanisms have been introduced, claiming a smooth enamel topography following stripping. The aim of this *in vitro* study was to quantitatively assess the changes in enamel roughness after interproximal stripping with oscillating diamond strips. The null hypothesis was that stripping does not increase the amplitude, hybrid, and functional roughness parameters of the enamel.

## Methods

### Sample preparation

Sixteen premolars devoid of caries, enamel cracks, fluorosis, or abrasion under stereomicroscopic inspection (M80, Leica, Wetzlar, Germany) at ×10 magnification were used in the study. The teeth had been extracted for orthodontic reasons, cleaned, and stored in distilled water with 0.5% sodium azide at 8°C.

In order to simulate intraoral stripping conditions, four teeth were embedded up to their cervical region in an addition-type polyvinylsiloxane putty impression material (Aquasil Putty, Dentsply/Detrey, Konstanz, Germany), creating thus an arch with three interproximal contact areas. Four equal setups were used (*n* teeth = 16, *n* contact points = 12, *n* sites measured = 24). Before embedding, the roughness parameters of the proximal untreated surfaces were measured and served as reference.

Grinding was performed according to the manufacturer's instructions under water cooling by one operator (SB). The three interproximal areas per setup were ground on both sides (mesial/distal) using the Ortho-Strips system (Intensiv Dental SA, Montagnola, Switzerland) employing two-sided flexible diamond-coated strips of three different grit sizes (40 μm for contouring, 25 μm for finishing, and 15 μm for polishing). New devices were used for each stripping session. The strips used were attached to a micromotor (Eva Intra Lux Prophy head 61 LRG KaVo, Biberach, Germany, 7,500 rpm) operated for 20 s per grit size. Under these conditions, a 0.25-mm interproximal enamel reduction was achieved as checked by a steel ligature. The roughness parameters of the stripped areas were measured again under the same conditions.

### Roughness parameter measurements

Roughness analysis was performed by optical interferometric profilometry. A 3D optical profiler was used (Wyko NT 1100, Veeco, Santa Barbara, CA, USA) under the following conditions: vertical scanning mode, Mirau lens, ×20.3 magnification (231.1 × 303.8 μm^2^ analysis area), tilt correction, 5-μm Gaussian high-pass filter, and 0.2 μm (*x*, *y*) and 0.1 nm (*z*) resolution. Three measurements were performed per specimen and averaged representing surface roughness before and after stripping at the same region per tooth. The roughness parameters tested were the following:

- The amplitude parameters Sa (the absolute profile deviation versus the average over a 3D surface) and Sz (the ten-point height over the complete 3D surface representing the average difference between the five highest peaks and five lowest valleys)
- The hybrid parameter Sdr (the developed interfacial area ratio, expressed as the percentage of additional surface area contributed by the texture as compared to an ideal plane of the same size)
- The functional parameters Sci (the core fluid retention index, a measure of the volume the surface would support from 5% to 80% of the bearing ratio) and Svi (valley retention index, the volume the surface would support at the valley zone, 80% to 100% of the bearing ratio)

The differences (Δ: after-before stripping) of the individual roughness parameters were calculated per tooth and region [16].

### Statistical analysis

The results of ΔSa, ΔSz, ΔSdr, ΔSci, and ΔSvi were statistically analyzed by one-sample Mann-Whitney rank sum test [17] at a 95% confidence level (*a* = 0.05).

## Results

**Figure 1:**
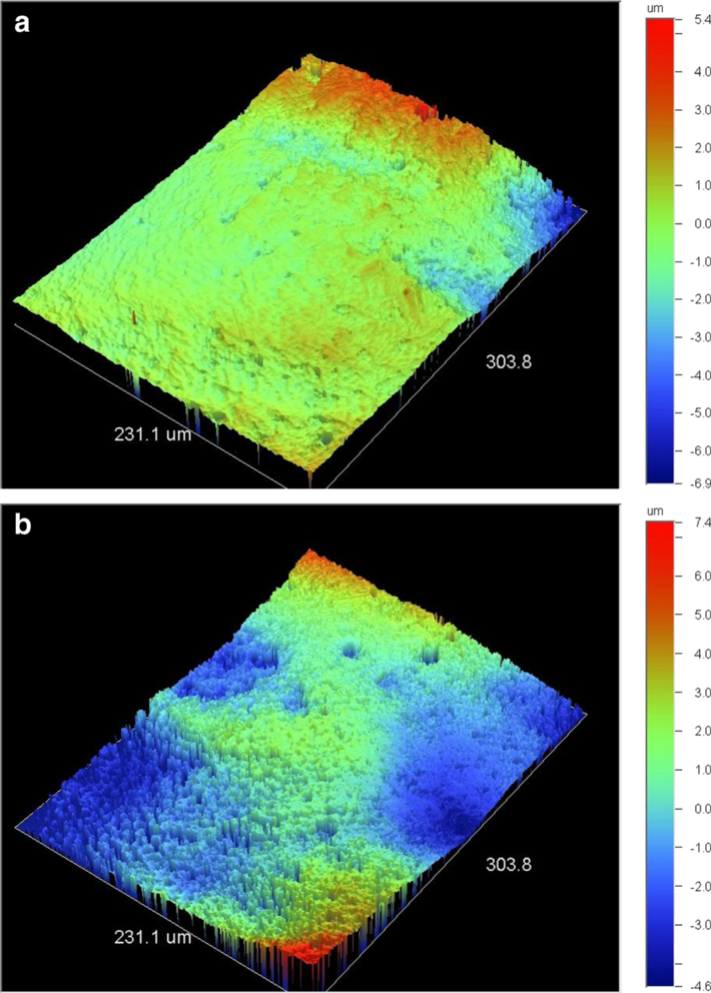
**3D profilometric images of enamel interproximal areas before stripping (×20.3). (a)** Area with a smooth topography; **(b)** distal area revealing a prismatic structure at the left/front region of the image.

**Figure 2:**
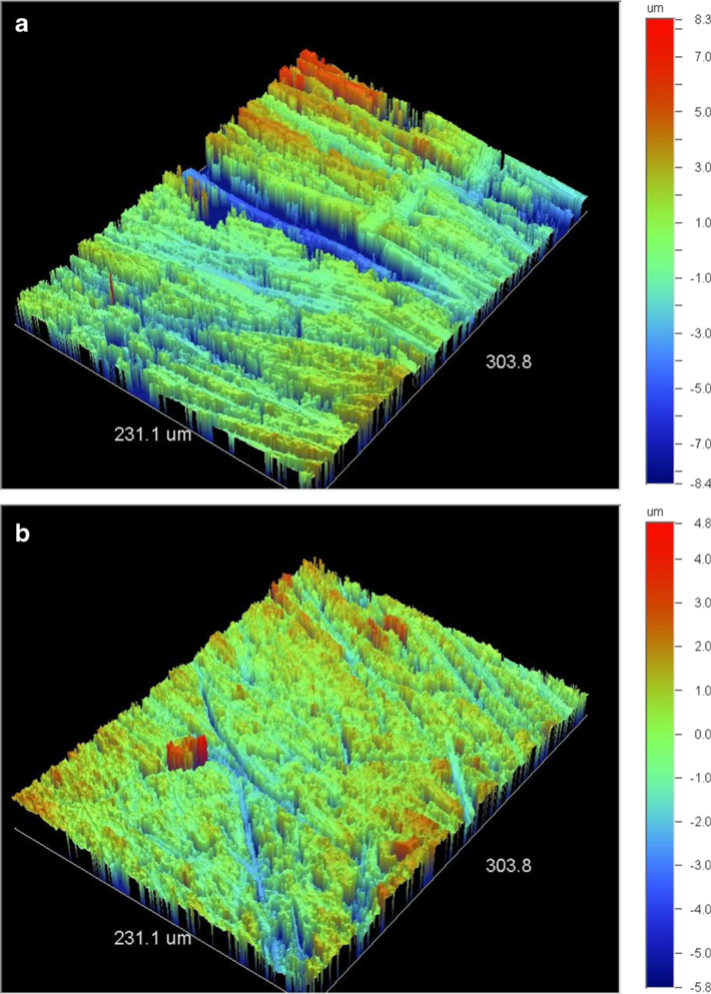
**3D profilometric images of enamel interproximal areas after stripping (×20.3). (a)** Mesial and **(b)** distal areas of the same teeth as in Figure 1. Both images reveal increased roughness. Note the orientation of the grooves produced from the grinding direction of the strips.

Representative 3D profilometric images of interproximal enamel margins before stripping are illustrated in Figure 1a,b. At the beginning, the enamel surface is smooth with no specific features, apart from the appearance of a prismatic structure in some domains (Figure 1b). After stripping (Figure 2a,b), the same regions presented a rough texture with polishing grooves produced from the grinding direction of the strips.

The results of the differences in the surface roughness parameters (Δ: paired values of the same region after-before stripping) and the results of the statistical analysis are presented in Table 1. The Mann-Whitney rank sum test showed very high statistically significant differences (*p* < 0.001) in ΔSa, ΔSz, and ΔSvi; a statistically significant difference (*p* < 0.05) in ΔSdr; and no statistically significant difference (*p* > 0.05) in ΔSci.

**Table 1:** **Statistics for the five roughness parameters tested**

	**Median value**	**25th percentile**	**75th percentile**	**Range**	**Max**	**Min**	***T*** **statistics**	***p*** **value**
ΔSa (μm)	0.453	0.075	0.710	2.940	2.000	−0.940	756	<0.001
ΔSz (μm)	3.870	0.340	5.500	17.720	11.320	−6.400	420	<0.001
ΔSdr (%)	1.514	−0.155	2.640	11.770	8.200	−3.500	708	0.014
ΔSci	0.110	−0.145	0.325	2.030	1.450	−0.580	648	0.219
ΔSvi	0.040	0.08	0.060	0.130	0.100	−0.030	768	<0.001

## Discussion

Interdental stripping is a commonly used technique in orthodontics that provides important clinical advantages, but with several limitations. Enamel reduction may impair tooth resistance against the aggressive intraoral environment and may lead to sensitivity [18] or caries development due to plaque accumulation [11-13].

Controversial evidence has been reported so far on the effect of stripping on enamel morphology and roughness. SEM studies [19,20] showed that perforated diamond-coated disks with less than 30-μm grain size minimized grinding defects, whereas subsequent polishing with fine and extra-fine alumina disks (Sof-Lex, 3M ESPE, St. Paul, MN, USA) produced smooth surfaces, equally to or smoother than untreated enamel. Nevertheless, the results of this study were mainly based on subjective qualitative assessment of enamel morphological characteristics. On the other hand, qualitative assessment by SEM and quantitative assessment of the amplitude parameter Ra by in-line stylus profilometry [21] showed that stripping disks and diamond-coated metal strips followed by fine Sof-Lex disks produced significantly rougher surfaces in comparison with the intact enamel of permanent and deciduous teeth. Qualitative (scanning electron microscopy) and quantitative (surface roughness test) measurements by Gupta et al. [22] also showed that the enamel after stripping with diamond disks and different polishing methods was significantly rougher than untreated control teeth. To improve the reliability of quantitative analysis, 3D optical profilometry was used to determine the Ra values by scanning sample areas rather than performing in-line analysis on enamel areas [23]. The results showed that grinding and polishing with automatic oscillating systems, including Ortho-Strips, resulted in equally smooth surfaces with untreated enamel, or even better. These systems are considered to provide better results than other common stripping techniques, where enamel defects have been observed [12,14,24].

The results of the present study failed to confirm the findings of the aforementioned studies for the Ortho-Strips system regarding the Sa amplitude parameter (the 3D equivalent to Ra). Moreover, all the parameters tested in the present study (amplitude, hybrid, functional) showed significantly increased values after stripping, except for Sci. Therefore, the null hypothesis should be partially rejected.

As previously considered [23], only the quantitative assessment of roughness parameters allows direct comparisons among surface treatments, without the subjective assessment bias of the SEM techniques. Since optical interferometric profilometry is a non-destructive technique, it may be sequentially used on the same specimens before and after stripping. In such experimental designs, as in the present study, measurements are taken at the same region of the same tooth before and after stripping, the values before stripping serving as controls. Therefore, the variability in roughness values of a separate control group is neutralized by expressing the difference (Δ) induced in surface roughness parameters at the same region of each individual specimen and compares the difference versus the zero value [17]. To further standardize the procedure, a ×20.3 magnification was used to analyze more enamel areas, whereas three regions were measured and averaged per surface and treatment to assure reproducibility. This experimental design provided a total number of 24 measurements for each condition and parameter, which resulted in an equal number of roughness value differences for evaluation.

An important issue, frequently overlooked, is that for the reason of comparison with previous literature, roughness parameters are limited only to Ra (in 2D) or Sa (in 3D) measurements. Although popular, Ra and Sa quantify the ‘absolute’ height or amplitude of the surface peaks or valleys (all considered as peaks) and are insensitive to their spatial distribution [16,25]. This is the reason for including three types of roughness parameters that were evaluated: amplitude, hybrid, and functional. In the amplitude parameters, besides the common Sa, the Sz was also included. Sz can discriminate between peaks and valleys, being more sensitive to Sa when studying wear effects, just like the stripping-induced effects on enamel. Sdr is related to slope sizes and provides information on the additional surface area produced from the surface texture in comparison with an ideal plane surface of the same size. Sci is associated with the relative retention of fluid the core surface structure provides, and Svi is linked to fluid retention at deepest valleys [16].

The results of the present study showed statistically significant differences in ΔSa, ΔSz, ΔSdr, and ΔSvi and no difference in ΔSci. The statistically significant difference found in the amplitude parameters Sa and Sz implies that the enamel surface left after stripping has higher peaks or/and deeper valleys in comparison with its native reference (intact enamel surface). This was clearly observed in the 3D profilometric images. Since increased amplitude heights (positive or negative) affect the peak and valley slopes, the enamel surface area is increased, a fact confirmed by the statistically significant difference found in ΔSdr. The absence of statistically significant differences in ΔSci implies that the core surface structure of enamel after stripping (excluding the 5% of the shallowest and the 20% of the deepest valleys as per Sci definition) provides the same fluid retention capacity as the intact control. However, in the Svi index, where the contribution of the 20% of the greatest valleys at the bearing ratio is taken into account, a statistically significant difference was found which could substantially increase the plaque retention capacity.

The differences in the profilometric methodology of the present study from the previous were as follows: a) The same surfaces of the specimens used as controls (intact enamel) were scanned before and after stripping (sequential treatment mode). This provides important advantages over the use of another series of specimens as a control group, since the same region is tested before and after in the sequential mode. b) More roughness parameters were tested to better characterize the enamel surfaces. The need for further polishing became evident from the results of the study. However, it is extremely difficult to use other types of interproximal polishing when the proximal reduction is 0.25 mm. Hand-operated finishing strips (i.e., for composite restorations) with a thickness of 0.10 to 0.15 mm may be used, but fail to fully adapt to the curved surface. Common Sof-Lex disks (stiff, urethane-backed/3M ESPE) have a greater thickness than the proximal reduction (approximately 0.28 vs 0.25 mm of the reduction) and therefore cannot be used. Extra-thin Sof-Lex disks (flexible, polyester-backed/3M ESPE) may fit, but they are very flexible and need a high speed for final polishing, putting at risk the integrity of marginal soft tissues. The results highlight the finding that the surface left after stripping with the Ortho-Strips system is rougher than native enamel, contrary to what has been stated in the literature so far.

Application of stripping in clinical conditions involves teeth, which have the ability to move as the pressure applied by the machine is partially absorbed by the periodontal ligament displacement. Therefore, the lack of stiffness of the system may reduce the amount of enamel tissue ground. The design of the present study included embedding of teeth in a silicone substrate for limited tooth movement in an attempt to simulate the clinical condition.

It has long been established that amplitude parameters alone cannot properly describe the roughness of a surface [26]. Actually, for a complete characterization of the roughness of a surface, 15 parameters are required, including 4 amplitude, 4 spatial, 4 hybrid, and 3 functional [16]. Therefore, there is a need for a concise characterization of the enamel surface roughness parameters including amplitude, spatial or hybrid, and functional parameters. Such an approach would make comparisons among treatments more feasible and establish a basis for explanation of the biological responses.

## Conclusions

Summarizing the results of the present study, enamel surfaces after interproximal grinding and polishing with the Ortho-Strips system showed statistically significant increase in Sa, Sz, Sdr, and Svi roughness parameters from their native intact controls, but no statistically significant differences in Sci.

The statistically significant difference found in the amplitude parameters Sa and Sz implies that the enamel surface left after stripping has higher peaks or/and deeper valleys in comparison with its native reference.
